# The unveiling of a new risk factor associated with bladder cancer in Lebanon

**DOI:** 10.1186/s12894-019-0445-9

**Published:** 2019-03-05

**Authors:** Sally Temraz, Yolla Haibe, Maya Charafeddine, Omran Saifi, Deborah Mukherji, Ali Shamseddine

**Affiliations:** Department of Internal Medicine, American University of Beirut Medical Center, P.o.Box: 11-0236, Riad El Solh, Riad El Solh, Beirut, 110 72020 Lebanon

**Keywords:** Bladder cancer, Smoking, Trihalomethanes, Lebanon, Water pollution

## Abstract

**Background:**

No accurate evaluation of smoking and water pollution on bladder cancer has been conducted in the Lebanese population. Our aim is to examine the significance of smoking and one of the main water pollutants Trihalomethanes (THM) on bladder cancer risk.

**Methods:**

Population Attributable Fraction (PAF) was used to quantify the contribution of the risk factors smoking and THMs on bladder cancer in Lebanon. To calculate PAF for each risk factor, we used the proportion of the population exposed and the relative risk for each risk factor. Relative risks for each risk factor were obtained from published meta-analyses. The population at risk values were obtained from a report on chronic disease risk factor surveillance in Lebanon which was conducted by the World Health Organization between 2008 and 2009 and a national study by Semerjian et al. that conducted a multipathway exposure assessment of selected public drinking waters of Lebanon for the risk factors smoking and THMs, respectively.

**Results:**

Bladder cancer cases that were the result of smoking in Lebanon among males and females are 33.4 and 18.6%, respectively. Cases attributed to mid-term exposure to THM contamination of drinking water is estimated at 8.6%.

**Conclusion:**

This paper further highlights the negative impact of smoking on bladder cancer risk and adds an overlooked and often underestimated risk that THMs have on this type of cancer. Thus, it is imperative that a national based study which assesses THM exposure by gender and smoking status be implemented to determine the real risk behind this byproduct.

## Background

Bladder cancer is the ninth most common cancer among both sexes combined, accounting for 3.1% of all cancer cases in the world [1]. Incidence rates vary from 10 to 30 cases/100,000 for men and 1–6 cases/100,000 in women [2]. In addition to the well-known risk factors including advanced age, male gender and white race, environmental risk factors have significantly influenced incidence rates among exposed individuals [2, 3]. The International Agency for Research on Cancer (IARC) identified smoking as one of the causes of bladder cancer in both men and women [4]. Moreover, two large cohort studies conducted in Europe and the USA found that cigarette smoking is an important risk factor for bladder cancer [5, 6]. Another potential risk factor for cancer is the production of chlorination by-products which are introduced during water treatment [7]. Naturally occurring humic and fluvic acids in water from decomposed plant and organic chemical interact with chlorine to form hundreds of halogenated chemical species, including trihalomethanes (THM), other haloalkanes, haloalkenes, haloacetic acids, other haloacids, haloacetonitriles, haloketones, haloaldehydes, and others [8]. The most commonly found and studied are trihalomethanes, with the highest concentration of chloroform, this chemical interaction occurs at the first stage of chlorination until distribution. THMs and other haloacetate byproducts were found carcinogenic in animal models and mutagenic in bacterial systems [8]. Two meta-analyses reported an association between THMs ingestion and bladder cancer risk [9, 10] and several case-control studies supported this association [11–14]. Although some reported a modest association via the ingestion route [14], the main discrepancies in results were related to those reporting an association between THMs exposure through shower, swimming pools and inhalation and bladder cancer risk [15, 16]. The IARC classified THMs as Group 3 carcinogens concluding that there was inadequate evidence for their carcinogenicity to humans from the ecological and death certificate studies.

Recent data from the Lebanese National Cancer Registry (NCR) revealed that bladder cancer was the second most common cancer in males in 2008 after prostate. In fact it has the highest incidence rate in the Arab World, reaching 34 cases per 100,000 [1, 17]. This rate is expected to increase to 41/100,000 by 2018 considering the increasing number elderly and the high smoking levels [17].

Smoking remains the most well established risk factor in respiratory cancers and bladder cancer. Additional risk factors including water pollution have been studied to explain the rise in bladder cancer in specific areas among non-smokers. However, no accurate evaluation of smoking and water pollution on bladder cancer has been conducted in the Lebanese population. Our aim is to examine the significance of smoking and water pollution on the incidence of bladder cancer in Lebanon.

## Methods

In this retrospective data analysis, the exposure to the risk is examined in relation to bladder cancer occurrence. The population attributable fraction (PAF) was used to estimate the role of THM on bladder cancer incidence in Lebanon. To quantify the contribution of a risk factor to cancer, two main components are needed; the proportion of the population subjected to the risk and the relative risk of cancer associated with that risk factor. Cancers that are the consequence of exposure to a risk factor are usually the result of an accumulation to that exposure over many years; a latent period of 10 years is viewed as satisfactory for most risk factors [18].

### Prevalence of risk factors

In order to study the impact of smoking and water pollution on bladder cancer in the Lebanese population, we relied on population based reports or studies involving a representative sample of the Lebanese population that identifies the proportion of the population at risk. We used a report on chronic disease risk factor surveillance in Lebanon which was conducted by the World Health Organization between 2008 and 2009. The report involved subjects aged 25–64 years that were randomly selected using Lebanese Governorates strata [19]. From this report, data on behavioral factors such as tobacco smoking, physical activity and alcohol consumption were obtained as well as body mass index (BMI), age and gender [19]. We also studied the effect of the carcinogen THM found in water samples in Lebanon. For data on THM contamination of drinking water, we relied on a report published by Semergian et al. [20], where they found that an average of 94.7% of investigated networks in Lebanon exceeded the set United States Environmental Protection Agency (USEPA) range of concern for increased carcinogenic risk from THM. [21]. According to the Central Administration for Statistics (CAS), the only sources of drinking water are underground water or surface water. Thus bottled water, which is derived from either ground or surface water, is a representation of both.

To complete the analysis, relative risks were retrieved for each risk factor from meta-analyses from epidemiological studies, as shown in Table 1.

**Table 1:** Showing population at risk and relative risk for each risk factor associated with bladder cancer in Lebanon

Risk factor	Population at risk		Reference	Relative risk		Reference
Smoking	Males	Females	[19]	Males	Females	[22]
Ex-smokers	6.9%	3.3%		1.5	0.92	
Current smokers	46.8%	31.6%		2	1.73	
Trihalomethanes	94.7%		[20]	1.1–1.4		[9, 20]

### Risk factor: Smoking

We estimated the proportion of bladder cancer cases that were the result of smoking (including smokers and previous smokers). Data were stratified as: smokers, ex-smokers and non-smokers for all age and gender categories. The role of smoking on bladder cancer was calculated for each age and gender separately, aggregating the smokers and ex-smokers in one category in the final analysis. Noting that the relative risk (RR) for smokers and ex-smokers differ, calculations for each category were carried out separately first.

The RRs for smokers and ex-smokers were retrieved from a large meta-analysis in Korea [22], in which the population smoking habits and behavior is similar to our population.

### Risk factor: THM

To obtain the RR of THM on bladder cancer risk, we relied on a study by Semerjian et al. that conducted a multipathway exposure assessment of selected public drinking waters of Lebanon based on the concentrations of THM within water distribution systems [20]. We also relied on a meta-analysis which involved six case-control studies (6084 incident bladder cancer cases, 10,816 controls) and two cohort studies (124 incident bladder cancer cases) [9]. The combined OR for mid-term exposure in both genders was 1.10 (95% CI 1.0 to 1.2) and for long term exposure was 1.4 (95% CI 1.2 to 1.7). Consistent results were shown in the Semergian study that noted an increased cancer risk for the spring season ranging between 1.19–1.39 folds [20]. The meta-analysis used a combination of RR and ORs. Noting that when the incidence of the outcome is low, such as in this case, it is safe to assume that the OR reported by Villanueva et al’s metanalysis is equal to the RR.

### Statistical method

To estimate the contribution of a risk factor to disease burden, this is expressed in the percentage of disease that is caused by a specific risk factor. The attributable risk in a population depends on the prevalence of the risk factor and the strength of its association (relative risk) with the disease. The formula is

$$ \mathrm{PAF}=\mathrm{Pe}\ \left(\mathrm{RRe}\hbox{-} 1\right)/\left[1+\mathrm{Pe}\ \left(\mathrm{RRe}\hbox{-} 1\right)\right] $$

where Pe is the prevalence of the exposure and RRe is the relative risk of bladder cancer due to that exposure.In the case where the risk factor presents more than one exposure level, such as in tobacco smoking (ex-smokers, current smokers), a modified formula of the Levin formula was adopted as follows [23]:

$$ \mathrm{PAF}={\mathrm{P}}_1\left({\mathrm{RR}}_1\hbox{-} 1\right)+{\mathrm{P}}_2\left({\mathrm{RR}}_2\hbox{-} 1\right)/1+\left({\mathrm{P}}_1\left({\mathrm{RR}}_1\hbox{-} 1\right)+{\mathrm{P}}_2\left({\mathrm{RR}}_2\hbox{-} 1\right)\right) $$

We thus applied this formula to obtain the PAR for each of the above mentioned risk factors.

## Results

We applied the formula for each of the risk factors and results are reported in Table 2.

**Table 2:** Percentage attributable risk of smoking, THM on bladder cancer cases in Lebanon

Formula application	Gender	25–34	35–44	45–54	55–64	ALL
Smoking	M	29.5%	34.3%	37.5%	34.7%	33.4%
	F	11.2%	19.2%	24.7%	24.2%	18.6%
Trihalomethanes	Both	NA	NA	NA	NA	8.65%

*NA* not available

- Smoking:
  By applying the modified Levin formula, we used as P_1_ the percent of males that were current smokers (46.8%) and RR_1_ the relative risk due to smoking which is 2 and used as P_2_ the percent of males that were ex-smokers (6.9%) and RR_2_ the relative risk due to ex-smoking which is 1.5. Bladder cancer cases that were the result of smoking in Lebanon among males were 33.4% of cases for all age groups. Similarly, we applied the formula for females and found that bladder cancer cases that were the result of smoking among females were 18.6% of cases for all age groups if we consider the smokers percentage in 2009 and its implication by 2018. The risk impact is not immediate; we need a 10-year lag to draw any conclusions. For specific age groups of males and females, the percent of smokers and ex-smokers for each age category were obtained from the WHO’s Chronic Disease Risk Factor Surveillance data for Lebanon [19] and formula applied. Results are shown in Table 2.
- THM contamination:
  Chlorination is the most adopted method of water sanitization in Lebanon, it is economical, quick and accessible. To calculate the percentage of bladder cancer cases attributed to mid-term exposure to THM contamination of drinking water we used the value of 94.7% for the Pe and value of 1.1 for RR since exposure to THM in Lebanon is midterm exposure. By applying the formula, we found that around 8.6% of bladder cancer cases in Lebanon could be attributed to THMs.

## Discussion

Our results reveal that the proportion of bladder cancer cases in the Lebanese population which could be attributed to smoking are 33.4% in males and 18.6% in females. The prevalence of smoking in Lebanon is on the rise where adult smoking is estimated at 38.5% (males at 46% and females at 31%) and youth smoking is highest worldwide (65.8% for boys and 54.1% for girls) [24], with waterpipe smoking the major form of smoking (33.9%) followed by cigarette smoking (8.6%) [24]. Cancer incidence studies and the NCR consistently showed that bladder cancer has been distinctively high compared to regional and international rates [17, 25, 26]. We estimate that over a hundred case control and cohort studies have evaluated the risk attributed to smoking on bladder cancer. An analysis performed by Freedman et al. in 2011 which included 467,528 men and women found that compared to non-smokers, former and current smokers had a two- and a four-fold increased risk of bladder cancer, respectively [6]. The population risk of bladder cancer attributable to smoking was approximately 50% for both men and women [6].

Chlorination of water before it reaches the consumer is highly prevalent in Lebanon and our results show that THM contamination of drinking water could be responsible for 8.6% of bladder cancer cases. Initially, water chlorination played an essential role in reducing mortality rates due to water contamination with pathogens. Chlorination was seen as a miraculous public health solution in the early twentieth century when concerns were restricted to the presence of pathogens in water. The first potential hazards of chlorination were introduced in 1974 linking a toxic byproduct of this chlorination with organic matter to cancer risk [27]. The summary of the results of a meta analysis that evaluated the association between chlorinated drinking water and different cancer sites showed that it only affects the bladder (*P* < 0.001) and rectum (*P* = 0.04), whereas it showed no significant results in all of other sites including gastrointestinal sites, breast, and respiratory sites [7].

Our analysis was calculated for mid-term exposure since chlorination has not been used for a long time in Lebanon. However, within 20 years, the exposure would be long term and the risks are anticipated to be higher. A meta-analysis investigating the role of long term exposure of over 40 years to chlorinated drinking water in six case-control studies, revealed that it was associated with an increased risk of bladder cancer in men (combined OR = 1.4, 95% CI 1.1 to 1.9) and women (combined OR = 1.2, 95%CI 0.7 to 1.8, 9]. Another meta-analysis by Costet et al. reported a significant odds-ratio for men exposed to an average residential THM level > 50 μg/L (OR = 1.47) when compared to men exposed to levels ≤5 μg/L. The risk was not significant in females or through cumulative exposure through ingestion [10].

In addition to water ingestion, the high volatility and permeability of some disinfection by-products suggests that they can get into human bodies through bathing, washing vegetables, and swimming [27]. Following the solid waste management and landfill scandal in Lebanon, marine pollution gained major media attention and warned swimmers of accessing coastal areas in the summer. This news led a large proportion of swimmers to escape sea water pollution and opt for resort swimming pools instead. However, it was revealed that the chlorine concentration levels in these pools are high and uncontrollable as it is added arbitrarily in any of the private beach resorts thus increasing the exposure of the people to THM.

A case-control study conducted in Spain by Villanueva et al. tackling all entry routes of THMs, showed that individuals with long term exposure to THMs were twice as likely to have bladder cancer, OR = 2.10 (95% confidence interval: 1.09, 4.02) for average household THM levels of > 49 versus < 8 μg/L. When compared to controls, subjects who were drinking chlorinated water with a total THM exposure > 35 μg/day had an odds ratio of 1.35 (95% CI: 0.92, 1.99). On the other hand, the odds ratio attributed to exposure to highest compared to lowest quartile THM exposure through duration of shower or bath was calculated at 1.83 (95% CI: 1.17, 2.87). The odds ratio attributed to swimming in pools was 1.57 (95% CI: 1.18, 2.09) [28]. On the other hand, a recent case control study by Freeman et al. reported significant adjusted ORs for bladder cancer comparing participants with exposure above the 95th percentile with those in the lowest quartile of daily exposure to brominated THMs [OR = 1.98 (95% CI: 1.19, 3.29), *p* - trend = 0.03] and cumulative exposure to brominated THMS 1.78 (95% CI: 1.05, 3.00), *p* - trend = 0.02 but was not significant for chlorinated THMs. Moreover, Freeman et al. did not find any association between swimming pool use and bladder cancer contrary to what Villanueva et al. reported. Thus, the association between THMs and bladder cancer risk is still inconclusive but seems to be more in favor of the ingestion route of THMs than exposure through skin.

Several limitations in our study should be noted. First, we have no national based data on THM concentrations in drinking water as we have for smoking. This deficiency of exposure to THM by age, and gender limited our calculation to a general estimate of the exposure for all population. Socio economic and geographical differences were not accounted for while they can affect drinking water availability and exposure to contaminated water. Our results are therefore general estimates and not an actual representation of the water quality in Lebanon. Moreover, the risk calculated for THM included both smokers and non-smokers since our data source had limited information on the subject and did not stratify according to smoker and non-smokers. Although estimates, the figures reported reveal a possible problem in the drinking waters of Lebanon and risk of bladder cancer which was not previously accounted for. Our underground waters have been compromised with sewage and solid waste. It is thus imperative that a national based study be conducted to assess all sources of drinking water in Lebanon and a framework be placed to prevent further contamination and corrective measures be taken where possible.

## Conclusions

Our results have highlighted the impact of smoking on bladder cancer risk as have many previous reports all over the world. In our report, there were 33.4% of male cases of bladder cancers and 18.6% of female cases attributed to smoking. This paper adds an overlooked risk affecting bladder cancer that is the impact that THMs and have on bladder cancer risk. We estimated the risk from THM exposure to be 8.65%. A national based study to assess THM exposure by gender and smoking status is highly needed to determine the risk of this contaminant on bladder cancer.

## Abbreviations

BMI: Body mass index
CAS: Central Administration for Statistics
CC: Chronic cystitis
IARC: The International Agency for Research on Cancer
LARI: Lebanese Agricultural Research Institute
NCR: National Cancer Registry
PAF: Population attributable fraction
TCC: Transition cell cancer
THM: Trihalomethanes
USEPA: United States Environmental Protection Agency
